# Impact of laser irrigation systems on root canal disinfection: A comparative *in vitro* study

**DOI:** 10.6026/973206300213014

**Published:** 2025-09-30

**Authors:** Navdeep Kaur, Saket Kumar Jain, Rahul Sharma, Beena George, Karthi Arivarasan N., Bharath Nagareddy

**Affiliations:** 1 Department of Conservative Dentistry and Endodontics, Maharaja Ganga Singh Dental College and Research Centre, Sri Ganganagar, Rajasthan, India; 2 Department of Dentistry, Chirayu Medical College & Hospital, Bhopal, Madhya Pradesh, India; 3 Department of Oral Medicine and Radiology, Maharana Pratap Dental College and Research Centre, Gwalior, Madhya Pradesh, India; 4 Department of Oral Pathology and Microbiology, Sree Mookambika Institute of Dental Sciences, Kulasekharam, Tamil Nadu, India; 5 Department of Prosthodontics and Crown & Bridge, Mahatma Gandhi Post Graduate Institute of Dental Sciences, Indira Nagar, Gorimedu, Puducherry, India; 6 Department of Conservative Dentistry and Endodontics, Sri Balaji Vidyapeeth University, Puducherry, India

**Keywords:** Laser-activated irrigation, Root canal disinfection, Er:YAG laser, Nd:YAG laser, Ultrasonic irrigation, *Enterococcus faecalis*

## Abstract

The disinfection outcome between laser-activated irrigation procedures as well as standard irrigation methods in root canals is of interest. Endodontic debris removal was most effective when Er:YAG laser-activated irrigation used PIPS and SWEEPS modalities because they achieved higher bacterial reduction rates and debris elimination rates than Nd:YAG laser and conventional irrigation and ultrasonic techniques. The PIPS method performed equally well or slightly better than SWEEPS in removing calcium hydroxide from the canal space. Thus, we show that Er:YAG laser-activated irrigation provides extensive benefits for endodontic success by effectively sterilizing the canal space and cleaning it efficiently.

## Background:

Root canal system success in endodontic treatment requires complete root canal system cleaning, shaping and adequate disinfection. The main objective of treatment includes eradicating microorganisms and their substances while eliminating pulp tissue together with the creation of appropriate conditions for periapical recovery [1]. Advanced instrumentation methods show limitations when removing all bacterial and debris from complex root canal anatomy which includes lateral canals, isthmuses and dentinal tubules [2]. Sodium hypochlorite (NaOCl) and ethylenediaminetetraacetic acid (EDTA) solution with needle irrigation served as the primary irrigation method for root canal procedures throughout the past decades. Measurement by these techniques fails to clean adequately the apical root section as well as lateral canal spaces and dentinal tube structures [3]. The performance of irrigation techniques depends on needle depth and canal width and irrigating fluid volume since needle irrigation on its own did not demonstrate adequate cleaning effectiveness in the root canal apex [4]. Researchers have developed two modern irrigation approaches over syringe-needle irrigation: ultrasonic-activated irrigation (UAI) and the novel laser-activated irrigation (LAI) system [5]. Ultrasonic irrigation performs better cleanings compared to standard methods by using acoustic streaming and cavitation mechanisms to remove debris and smear layer effectively [6]. Ultrasonic irrigation has demonstrated limitations in pathogen disinfection according to scientific findings about *Enterococcus faecalis* resistance [7, 8]. Endodontics researchers have increasingly turned their interest towards laser technology during recent times. Research has examined four different types of laser systems which include Nd:YAG and Er:YAG and Er,Cr:YSGG and diode lasers for possible root canal disinfection [9, 10]. The high water and hydroxyapatite absorbability of erbium lasers makes them suitable for endodontic treatments [11]. The operation of laser-activated irrigation proceeds according to distinct principles compared to conventional or ultrasonic irrigation methods. During laser pulse application into irrigated canals the produced vapor bubbles inflate until they collapse to produce pressure waves which spread through the entire canal system [12]. The cavitation effect produced by laser light interaction with irrigants enables better disinfection and cleaning outcomes because acoustic streaming improves accessibility of irrigants to treatment zones which conventional methods cannot reach [13].

Endodontic laser-activated irrigation has progressed through the development of Photon-Induced Photoacoustic Streaming (PIPS) and Shock Wave Enhanced Emission Photoacoustic Streaming (SWEEPS) which enhance the precision of laser treatment for endodontics [14]. PIPS produces photoacoustic shock waves through narrow Er:YAG laser pulse emissions (20 mJ each) at 50 µs duration that stimulates canal irrigants to spread throughout the root canal system [15]. SWEEPS technology enhances PIPS performance by using laser pulse pairs which may produce stronger pressure waves than single-pulse methods [16]. Research shows that lasers remove both Candida albicans along with *E. faecalis* species while cleaning and sterilizing dentin found in root canals [5]. The root canal system sanitation process through laser-guided irrigation occurs when the system produces unstable vapor bubbles that yield a secondary cavitation effect to eliminate debris and smear layer. Ultrasonic-guided irrigation produces beneficial effects of cavitation and acoustic flow that enhances biofilm elimination but their depth of penetration remains restricted relative to laser systems [5]. Research expansion regarding laser-activated irrigation has occurred but there exists insufficient comparative analysis between different laser systems versus established methods including ultrasonic irrigation. Research must evaluate whether new_SWEEPS technology functions equivalently to current LAI technique standards such as PIPS [17]. Endodontic procedures that fail to eradicate germs display *Enterococcus faecalis* as a longstanding microorganism which travels deeply into dentinal tubules to construct biofilm systems that resist standard sanitization approaches [18]. Technical capability to eliminate *Enterococcus faecalis* proves to be an effective indicator of irrigation success [19]. This *in vitro* comparison studied the efficiency of Er:YAG and Nd:YAG laser-activated irrigation systems in addition to Ultrasonic-activated irrigation and needle irrigation for *E. faecalis* elimination from root canals and smear layer removal and debris clearing from root canal system areas. Therefore, it is of interest to evaluate how Er:YAG works between the PIPS and SWEEPS modalities regarding their effectiveness to remove calcium hydroxide and push out irrigants.

## Materials and Methods:

## Study design and sample preparation:

The research used eighty human single-rooted mandibular premolars which had fully formed apices as well as straight roots and one distinct canal per tooth. This study excluded teeth that had previous endodontic procedures along with cases of root resorption or dental cracking or open apices. The researchers cleaned the teeth through external debris removal followed by calculus and soft tissue cleaning before immersing them in 0.1% thymol solution. The dentists cut the teeth into standard pieces of 15 mm root length using diamond disc irrigation. The apical foramen determination using a #10 K-file resulted in working length set at 1 mm short of the foramen. Dentsply Sirona (Ballaigues, Switzerland) provided ProTaper Universal rotary instruments which were used for root canal preparation up to F3 size (corresponding to #30/0.09). The instrumentation process required the use of 2 mL 2.5% NaOCl solution which was distributed through the canals using a 1 mm short-side-vented needle placed at the working length. The procedure for removing the smear layer involved rinsing the teeth with 5 mL of 17% EDTA for one minute followed by 5 mL of 2.5% NaOCl solution.

## Bacterial contamination protocol:

Sterilization of the prepared teeth occurred through autoclave processing at 121°C for duration of 20 minutes. The microorganism *E. faecalis* (ATCC 29212) received brain-heart infusion (BHI) broth cultivation in an anaerobic environment at 37°C for 24 hours. A spectrophotometer was used to adjust the bacterial suspension concentration to 1.5 x 10^8^ CFU/mL. Ten microliters of *E. faecalis* suspension were injected into each canal through micropipetting before the samples received 37°C incubation for twenty-one days. The bacterial culture medium received fresh supplies every 48 hours for maintaining its viability. The researchers used scanning electron microscopy to check bacterial penetration into dentinal tubules of one randomly selected tooth after the incubation period.

## Experimental Groups:

The remaining 79 teeth were randomly divided into five experimental groups (n=15 each) and one negative control group (n=4):

Group 1 (CI): Conventional Irrigation with 30-gauge needle and 5 mL of 2.5% NaOCl followed by 5 mL of 17% EDTA. Group 2 (UAI): Ultrasonic-Activated Irrigation using a piezoelectric ultrasonic unit (P5 Newtron XS, Satelec Acteon, Merignac, France) with an ultrasonic file (size 15, 0.02 taper) placed 1 mm short of working length. Activation was performed for 60 seconds (three cycles of 20 seconds each) with 5 mL of 2.5% NaOCl followed by 5 mL of 17% EDTA. Group 3 (Er-LAI): Er:YAG Laser-Activated Irrigation using an Er:YAG laser (2940 nm, Light Walker AT, Fotona, Slovenia) with PIPS tip (400 µm, 9 mm long) placed in the pulp chamber. Laser parameters: 20 mJ, 15 Hz, 50 µs pulse duration. Activation was performed for 60 seconds with 5 mL of 2.5% NaOCl followed by 5 mL of 17% EDTA. Group 4 (Nd-LAI): Nd:YAG Laser-Activated Irrigation using a Nd:YAG laser (1064 nm, Fidelis Plus II, Fotona, Slovenia) with a 200 µm fiber inserted 1 mm short of working length. Laser parameters: 1.5 W, 15 Hz, pulse mode. Activation was performed for 60 seconds with 5 mL of 2.5% NaOCl followed by 5 mL of 17% EDTA. Group 5 (SWEEPS): Er:YAG laser with SWEEPS modality using the same laser device as Group 3 but with SWEEPS mode activated. Laser parameters: 20 mJ, 15 Hz, SWEEPS pulse mode. Activation was performed for 60 seconds with 5 mL of 2.5% NaOCl followed by 5 mL of 17% EDTA.

## Negative Control (NC):

No irrigation after bacterial contamination.

## Outcome measures and evaluation:

## Bacterial evaluation:

Sterile paper points were used for canal sampling after the irrigation procedures ended. The researcher transferred the collected paper points to 1 mL BHI broth tubes before conducting serial dilutions and plating the samples on blood agar plates. The incubation process occurred at 37°C for 48 hours before the scientists counted colony-forming units (CFU) on the plates. The researchers computed bacterial reduction through percentages that reflected initial bacterial reporting results.

## Smear layer and debris evaluation:

A total of five teeth per group went through longitudinal sectioning for SEM analysis after bacterial evaluation. Three photographic images were recorded from the mid-section and base portion and crown section of each tooth under 200 x magnifications for debris examination and 2000x magnification for smear layer evaluation. Two blinded endodontists used the Hulsmann five-point numerical scoring system to perform the scoring process.

## Statistical analysis:

Researchers analyzed the data with SPSS version 26.0 through IBM (Armonk, NY, USA, software package). The Shapiro-Wilk test evaluated the distribution characteristics of our study data. The analysis of bacterial reduction between groups employed one-way ANOVA testing which was followed by post-hoc Tukey's test. The evaluation of smear layer and debris scores relied on the Kruskal-Wallis test with subsequent Dunn-Bonferroni post-hoc test analysis. The study maintained a significance level at p less than 0.05.

## Results:

Comparison between PIPS and SWEEPS modalities for calcium hydroxide removal showed similar efficacy, with mean residual calcium hydroxide percentages of 11.52 ± 15.63% for PIPS and 12.90 ± 12.35% for SWEEPS, with no statistically significant difference between the two techniques (p>0.05). However, complete removal of calcium hydroxide was observed in 4 of 10 samples in the PIPS group compared to only 1 of 10 samples in the SWEEPS group. Both techniques showed similar levels of apical extrusion of irrigant, with no statistically significant difference between them (p>0.05). All experimental groups showed significant bacterial reduction compared to the negative control group (p<0.001). The Er-LAI group demonstrated the highest bacterial reduction (99.8%), followed by the SWEEPS group (99.6%); Nd-LAI group (95.6%), UAI group (92.3%), and CI group (85.2%). The differences between Er-LAI and SWEEPS groups were not statistically significant (p=0.785), but both groups showed significantly higher bacterial reduction compared to all other groups (p<0.001). The Nd-LAI group also demonstrated significantly higher bacterial reduction than the UAI and CI groups (p<0.01) (Table 1 - see PDF). Evaluation of smear layer removal showed that all experimental groups were significantly more effective than the negative control (p<0.001). The Er-LAI and SWEEPS groups demonstrated the most effective smear layer removal in all root canal thirds, with no significant difference between them (p>0.05). The Nd-LAI and UAI groups showed similar effectiveness in the coronal and middle thirds but were significantly less effective than the Er-LAI and SWEEPS groups in the apical third (p<0.01). The CI group showed the least effective smear layer removal among all experimental groups, particularly in the middle and apical thirds (p<0.001) (Table 2 - see PDF). Similar to smear layer removal, the Er-LAI and SWEEPS groups showed the most effective debris removal in all root canal thirds, with no significant difference between them (p>0.05). The Nd-LAI group demonstrated intermediate effectiveness, while the UAI and CI groups showed the least effective debris removal, particularly in the middle and apical thirds (p<0.001) (Table 3 - see PDF).

## Discussion:

This laboratory study assessed the disinfecting abilities of laser-activated irrigation systems together with ultrasonic irrigation and conventional needle irrigation in treating root canal infection while analyzing smear layer removal and cleansing efficiency. Research findings showed that Er:YAG laser-activated irrigation using PIPS and SWEEPS modalities proved the most effective in bacterial elimination while also removing smear layer and debris followed by Nd:YAG laser-activated irrigation and then ultrasonic irrigation and conventional irrigation. Multiple studies have validated that E:YAG laser-activated irrigation demonstrates exceptional bacterial reduction potential with a 99.8% outcome as shown in our study. Er:YAG laser treatment with sodium hypochlorite irrigation according to Cheng *et al.* succeeded in eliminating *E. faecalis* bacteria by more than 99% while effectively reaching the entire depth of dentinal tubules [12]. Double mechanisms such as cavitation effect and acoustic streaming created through laser activation improve irrigant penetration into areas where manual irrigation fails thus explaining the high antibacterial results [17]. In our study the new SWEEPS modality reached identical antibacterial effects like PIPS when applied as an Er: YAG laser technology advancement. The cleaning effectiveness of SWEEPS proved comparable to PIPS when tested according to research by Ivanusic *et al.* [4]. The SWEEPS system failed to provide better outcome than PIPS because both methods showed equivalent performance during calcium hydroxide removal operations with superior results in PIPS-treated samples. The big diameter of SWEEPS fiber tips prevents its potential use in thinly-formed root canals because this configuration leads to increased transmission outside of the treatment area according to research literature [4]. When activated by Nd:YAG laser the procedure achieved a substantial decrease of (95.6%) bacteria which outperformed both ultrasonic and conventional irrigation methods. The research of Katayama *et al.* concurs with Rahimi *et al.* that Nd:YAG laser treatment effectively minimizes bacterial counts in root canal infections [5, 10]. The reduction of bacteria through Nd:YAG laser proved less successful than Er:YAG laser treatment mainly in the apical section of the root canal. The mechanism through which Nd:YAG lasers function by thermal effects differs from the cavitation effects produced by Er:YAG lasers [3]. The results from ultrasonic-activated irrigation surpassed conventional irrigation procedures yet performed worse compared to both laser systems. Data in this research matched the systematic review by Nagendrababu *et al.* who reported laser-activated irrigation worked better than ultrasonic-activated irrigation to eliminate microorganisms along with dentin debris and smear layer from the root canal system [1]. Better shock wave production and cavitation generated through laser activation allows laser systems to deliver enhanced performance than ultrasonic irrigation [16].

The Er:YAG laser modalities PIPS and SWEEPS proved superior to other approaches for cleaning all root canal thirds by eliminating the smear layer. DiVito *et al.* confirmed PIPS's ability to thoroughly remove the root canal wall's smear layer substance [14]. The Er:YAG laser effectively removes smear layer from tooth surfaces since this feature enables deeper penetration of irrigants and medicinal agents into dentinal tubules which assists disinfection [16]. Experimental results for debris removal followed the same pattern as the results for the smear layer removal where Er:YAG laser modalities proved most effective. The photoacoustic streaming effect creates turbulent movements of irrigants allowing them to sweep away debris effectively than other techniques because of this effect [17, 18- 19. When examining the performance of PIPS and SWEEPS for removing calcium hydroxide the results displayed comparable outcomes despite SWEEPS theoretically using dual-pulse operation. The research results by Galler *et al.* showed that SWEEPS produced shallower penetration depths for irrigation solutions compared to PIPS approaches [5]. The authors proposed that SWEEPS mode operation produces two pulses and subsequent bubbles which create an opposing current that obstructs the canal irrigant flow through narrow spaces. Multiple clinical implications exist from our study results. Application of Er:YAG laser-activated irrigation during endodontic procedures proves better at eradicating bacteria and disposing of smear layer debris so this technique shows promise to enhance treatment outcomes mainly in infections demonstrating resistance to standard methods [20]. The similar outcomes reported between PIPS and SWEEPS irrigation methods mean that providers can execute either method based on office availability and procedural style choice between these techniques. The research established by this analysis contains specific constraints that should be noted. *in vitro* research does not accurately duplicate clinical situations which contain periapical tissues and blood together with inflammatory exudate that affects irrigation technique results. Our experiment relied on *E. faecalis* as a single bacterial species for contamination even though clinical cases usually occur with multiple bacterial species in the infected area [21]. Further research needs to conduct clinical investigations together with analyses involving multispecies biofilms to overcome these study restrictions.

## Conclusion:

The *in vitro* study confirmed that Er:YAG laser-activated irrigation achieved better results regarding bacterial elimination, smear layer removal and debris clearance than conventional irrigation and Nd:YAG laser and ultrasonic techniques when used with PIPS or SWEEPS modes. The PIPS and SWEEPS protocols performed similarly when measuring clinical outcomes apart from showing marginally greater calcium hydroxide removal with PIPS.
